# Longitudinal associations between perceived social support and maternal depression and anxiety across the perinatal period

**DOI:** 10.1007/s00737-026-01785-0

**Published:** 2026-11-14

**Authors:** Mehreen Choudhary, Liesje Donkin, Amy Kercher

**Affiliations:** Department of Psychology & Neuroscience, Auckland University of Technology, Auckland, New Zealand

**Keywords:** Social support, Depression, Postpartum, Anxiety, Pregnancy, Longitudinal study

## Abstract

**Purpose:**

Despite robust international evidence that social support protects maternal mental health, the role of social support across the perinatal period remains poorly understood in Aotearoa New Zealand. This study aimed to examine how perceived social support relates to maternal depression and anxiety during the perinatal period, comparing partner, family, and friend support, and testing whether stress and sleep moderate these associations.

**Methods:**

Participants were 121 pregnant women who completed monthly surveys over 12 months, including the Edinburgh Postnatal Depression Scale, Perinatal Anxiety Screening Scale, and Multidimensional Scale of Perceived Social Support, along with measures of stress and sleep. Linear mixed models tested longitudinal associations and moderation effects.

**Results:**

Higher perceived social support was consistently associated with lower depression and anxiety. Family support was the most reliable protective source. Stress predicted higher distress and moderated the effect of support on anxiety, with support being more protective under high stress. Sleep problems predicted greater distress but did not moderate support effects.

**Conclusion:**

Findings suggest that perceived social support, particularly from family, is an important protective factor for maternal mental health. Screening for social support and stress in perinatal care may strengthen early identification and guide family-centred interventions.

**Supplementary Information:**

The online version contains supplementary material available at https://doi.org/10.1007/s00737-026-01785-0.

## Introduction

Pregnancy is a profound biopsychosocial transition marked by changes in physiological, emotional, cognitive, and social functioning. These changes often extend into the postpartum period and can leave women vulnerable to psychological difficulties (Bjelica et al. 2018). Depression and anxiety are among the most common mental health challenges during this time, with significant implications for both maternal wellbeing and child development (O’Hara and Wisner 2014). In Aotearoa New Zealand, elevated distress during the perinatal period is well documented, with Māori women reporting higher rates of depression and anxiety than non-Māori women (Signal et al. 2017). Low social support is one of the strongest and most modifiable predictors of perinatal distress (Bedaso et al. 2021; Harandi et al. 2017), yet questions remain about when, how, and for whom it is most protective.

Social support refers to perceived or actual assistance available through an individual’s network and plays a crucial role in psychological wellbeing (Cohen and Wills 1985). It comprises emotional, instrumental, and informational support (Schaefer et al. 1981). The stress-buffering hypothesis proposes that support may benefit wellbeing directly and mitigate the impact of stressors (Schaefer et al. 1981). Within stress and coping theory, social support functions as a key coping resource that alleviates distress when demands exceed internal capacity (Biggs et al. 2017; Lazarus and Folkman 1984).

Because this study was conducted in Aotearoa New Zealand, it is also important to consider Māori models of health that have shaped understandings of wellbeing and family-centred care within the New Zealand health context. While stress and coping theory offers valuable insight into maternal mental health, its foundations are grounded in Western, individualistic models. In contrast, Indigenous and Māori worldviews conceptualise wellbeing as embedded within collective relational systems. Concepts such as tūhono (bonding) and tūhonotanga (connectedness) describe attachment as grounded in whakapapa (genealogy), whānau (extended family networks), and spiritual interdependence (Mikahere-Hall 2020). Likewise, Durie’s (1994) Te Whare Tapa Whā, a Māori model of holistic health, positions whānau as a central dimension of health rather than an external coping resource. Together, these perspectives highlight why the source and continuity of social support may be particularly significant for maternal wellbeing within the sociocultural context of Aotearoa New Zealand.

Evidence shows that social support during pregnancy and postpartum is associated with lower maternal distress, although findings regarding timing and source remain mixed. Systematic reviews identify low support as a prominent risk factor for antenatal and postpartum depression and anxiety, but much of this literature is cross-sectional (Bedaso et al. 2021; Biaggi et al. 2016). Prospective studies show that low antenatal support predicts postpartum depression and that declines in support after birth increase risk even among previously well-supported women (Milgrom et al. 2008; Seymour-Smith et al. 2021; Żyrek et al. 2024).

Sources of support also shape outcomes. Partner support is frequently cited as the most protective source against postpartum depression (Milgrom et al. 2008; Yamada et al. 2020), yet its quality is as important as its presence, and women in unsupportive relationships may experience poorer mental health than those who are unpartnered (Bilszta et al. 2008). Family and friend support are also important, particularly for women who are unpartnered or in unsupportive relationships (Reid and Taylor 2015; Sufredini et al. 2022).

Stress is a well-established risk factor for perinatal depression and anxiety (van der Zee-van den Berg et al. 2021), yet evidence for stress-buffering effects of social support remains inconsistent, particularly for anxiety (Reid and Taylor 2015; Schwab-Reese et al. 2017; Yu et al. 2021). Longitudinal research suggests bidirectional links, with support predicting lower distress and distress eroding perceived support (Racine et al. 2019). Sleep disturbance is also a key correlate of perinatal depression and anxiety (Okun et al. 2018), and in Aotearoa New Zealand has been shown to compound maternal distress (Signal et al. 2022).

Although social support is well established as protective, important gaps remain. Few longitudinal studies have examined how changes in perceived support relate to both depression and anxiety across pregnancy and postpartum, particularly within Aotearoa New Zealand contexts (Hagaman et al. 2021; Li et al. 2017; Xie et al. 2009). Although partner support is often highlighted as most protective (Milgrom et al. 2008; Yamada et al. 2020), the relative contributions of family and friend support remain under-tested within the same models. In addition, few studies have examined whether social support moderates the effects of stress and sleep over time.

Using secondary analysis of longitudinal survey data from pregnant women in Aotearoa New Zealand, this study examined how perceived social support relates to depression and anxiety across pregnancy and postpartum, compared partner, family, and friend support within the same models, and tested whether stress and sleep moderate these associations.

## Materials and methods

### Study design and participants

This quantitative, longitudinal study involved secondary analysis of data from the Maternal Wellbeing and Exercise Through Pregnancy Study, conducted in Aotearoa New Zealand between 2023 and 2024. The original study examined maternal wellbeing across pregnancy and postpartum through repeated assessments of mental health, health behaviours, and psychosocial factors. Participants completed online questionnaires monthly for up to 12 months. The study was observational and did not involve any intervention.

Data were collected in two phases. A pilot phase (*n* = 34) assessed feasibility before a larger main phase (*n* = 87). Both phases used the same core psychological measures; however, the main phase included additional demographic and moderator variables (e.g. stress), and sleep items were subsequently harmonised. Recruitment for the main phase occurred via social media advertisements in community and parenting groups. Eligible participants were aged ≥ 18 years and pregnant. Following data cleaning, 11 participants were excluded because of ineligibility or missing baseline data, resulting in a final sample of 121 participants. Participants entered the study at varying gestational stages and completed repeated surveys across pregnancy and postpartum, resulting in unbalanced repeated measures.

The sample had a mean baseline age of 31.7 years (SD = 4.3) and a median gestational age of 24 weeks (IQR = 15–32). Participants predominantly identified as European/Pākehā, most were married or in a de facto relationship, education levels were generally high, and approximately two-thirds were employed full-time. Due to limited demographic data collected during the pilot phase, ethnicity, education, and marital status were unavailable for approximately 30% of participants and were treated as missing. Baseline sample characteristics are summarised in Table 1.

**Table 1:** Sample characteristics at baseline (*n* = 121)

Characteristic	*n* (%) or *M* (*SD*)	Range
Age (years)	31.7 (4.3)	18–40
Gestational weeks	23.8 (10.2)	4–52
Ethnicity		
European/Pākehā	62 (71.3%)	
Māori	12 (13.8%)	
Asian (South/East)	7 (8.0%)	
Pasifika	3 (3.4%)	
Other	3 (3.4%)	
Education		
Secondary school	14 (16.7%)	
Technical/trade	7 (8.3%)	
Undergraduate degree	34 (40.5%)	
Postgraduate degree	29 (34.5%)	
Marital status		
Married/de facto	83 (97.6%)	
Single	2 (2.4%)	
Employment status		
Full-time	79 (65.8%)	
Part-time	23 (19.2%)	
Not employed	18 (15.0%)	

Missing demographic data were present for approximately 30% of participants for ethnicity, education, and marital status; percentages are based on available data

### Measures

Depression, anxiety, and perceived social support were assessed using validated self-report instruments. Internal consistency of all multi-item measures was assessed using Cronbach’s alpha coefficients calculated from baseline responses.

#### Depression

Depressive symptoms were assessed monthly using the Edinburgh Postnatal Depression Scale (EPDS) (Cox et al. 1987), a 10-item self-report measure of symptoms over the past seven days (range 0–30). The EPDS demonstrates strong screening accuracy (Levis et al. 2020) and showed good internal consistency in this sample (α = 0.84).

#### Anxiety

Anxiety was measured at baseline, 3, 6, 9, and 12 months using the Perinatal Anxiety Screening Scale (PASS) (Somerville et al. 2014), a 31-item measure (range 0–93). Internal consistency in this study was excellent (α = 0.93).

#### Perceived social support

Perceived social support was assessed using the Multidimensional Scale of Perceived Social Support (MSPSS) (Zimet et al. 1988) at baseline, 3, 6, 9, and 12 months. The 12-item scale assesses support from partner (significant other), family, and friends using a 7-point Likert scale. Mean subscale and total scores (1–7) were analysed as continuous variables. The MSPSS demonstrates robust reliability. In the present sample, internal consistency was excellent for the total scale (α = 0.94) and subscales (α = 0.93–0.94).

#### Moderators

Stress and sleep were examined as potential moderators. Stressors were measured in the main study using a checklist of recent stressful life events (e.g. financial strain, relationship difficulties, unstable living conditions, health concerns). Items were summed to create a cumulative stressor count.

Sleep quality was assessed using Likert-type items that differed slightly between the pilot and main studies. Items assessed multiple aspects of maternal sleep, such as overall sleep quality (e.g. “How well have you been sleeping in the past week?”). Responses were harmonised into a binary indicator of sleep problems (present/absent).

### Data preparation

Datasets from pilot and main cohorts were merged and harmonised. Variables unavailable in the pilot cohort were retained as missing. Stressors were not measured in the pilot cohort; moderation analyses involving stress therefore primarily reflect the main sample.

Scale scores were computed according to manuals, with mean imputation applied where < 20% of items were missing.

Responses were classified by perinatal stage based on reported gestational or baby age (Pregnancy Trimester 1: 0–13 weeks, Trimester 2: 14–26 weeks, Trimester 3: 27–40 weeks or postpartum PP-Early: 0–12 weeks, PP-Mid: 13–25 weeks, PP-Late: 26–52 weeks). Where responses were missing, the nearest valid timepoint was used to estimate perinatal stage.

To examine patterns of attrition, baseline demographic and psychosocial variables were evaluated as predictors of survey completion. Higher baseline depression and anxiety, and lower education, were associated with fewer completed surveys, whereas no other baseline variables significantly predicted survey completion.

Data preparation was conducted in IBM SPSS Statistics for Windows (*Version 30.0*, 2024) and analyses in Jamovi (*Version 2.6.44*, 2025).

### Statistical analysis

Descriptive statistics summarised sample characteristics. Pearson correlations examined associations between perceived social support and distress at each stage.

Linear mixed models (LMMs) tested longitudinal associations between social support and symptoms of depression and anxiety, accounting for repeated measures. LMMs were selected because they use all available repeated observations and do not require complete data at every assessment. This approach is appropriate for longitudinal data with unbalanced follow-up. Analyses were conducted under the assumption that data were Missing At Random (MAR). Time was modelled as survey month or perinatal stage. Models examined total support, subscales, and moderation by stress and sleep. Random intercepts accounted for within-participant clustering.

Model assumptions were met, with no evidence of problematic multicollinearity. Partner support showed a negative skew consistent with a ceiling effect but did not affect model performance.

The final sample size (*n* = 121) with repeated observations provided adequate power to detect small-to-moderate effects (Maas and Hox 2005).

## Results

### Descriptive statistics by perinatal stage

Stage-level means for depressive symptoms (EPDS), anxiety (PASS), perceived social support (MSPSS), sleep problems, and stressor counts are provided in Table S1 (Online Resource 1). EPDS scores were highest during Trimester 1 and lowest in late postpartum, while PASS scores were highest in early postpartum and lowest in late postpartum. Perceived social support remained relatively stable, although friend support showed a slight decline during late postpartum. Sleep problems were most common in Trimester 3 and least common in Trimester 2. Mean stressor counts were low across stages.

### Correlations by perinatal stage

Pearson correlations between perceived social support and distress outcomes are reported in Table S2 (Online Resource 1). Higher perceived social support was generally associated with lower EPDS and PASS scores, with variation by source and stage. Family support showed the most frequent significant inverse correlations with EPDS and PASS from Trimester 2 through late postpartum. Partner support showed selective associations, more often with EPDS, while friend support reached significance less consistently. Full bivariate correlations are reported in Table S3 (Online Resource 1).

### Linear mixed models

#### Main effects of social support

Linear mixed models examined associations between perceived social support and maternal distress, adjusting for perinatal stage. Higher average total perceived support was associated with lower depressive (EPDS) and anxiety (PASS) symptoms, with no overall differences in symptoms between perinatal stages (Table 2, Panel A).

When examined separately, higher partner, family, and friend support were each associated with lower depressive symptoms, and partner and family support were also associated with lower anxiety. In combined models including all sources simultaneously, only family support remained a significant predictor of both depression and anxiety (Table 2, Panel B).

**Table 2:** Fixed effects from linear mixed models predicting maternal depression and anxiety from perceived social support (stage-based models)

Outcome	Model	Predictor	*b*	SE	95% CI [Lower, Upper]	*t*	*p*
Panel A: Total Perceived Support							
EPDS	Total	Total Support	-1.56	0.30	[-2.14, -0.98]	-5.29	< 0.001***
PASS	Total	Total Support	-3.55	0.70	[-4.92, -2.18]	-5.10	< 0.001***
Panel B: Sources of Support							
EPDS	Source-specific	Partner Support	-1.22	0.29	[-1.79, -0.65]	-4.24	< 0.001***
		Family Support	-1.02	0.21	[-1.43, -0.62]	-4.94	< 0.001***
		Friend Support	-0.58	0.22	[-1.02, -0.15]	-2.62	0.009**
	Combined	Partner Support	-0.60	0.35	[-1.29, 0.09]	-1.71	0.089
		Family Support	-0.73	0.25	[-1.22, -0.24]	-2.90	0.004**
		Friend Support	-0.22	0.23	[-0.67, 0.23]	-0.96	0.339
PASS	Source-specific	Partner Support	-2.75	0.66	[-4.05, -1.45]	-4.18	< 0.001***
		Family Support	-2.64	0.47	[-3.56, -1.71]	-5.63	< 0.001***
		Friend Support	-0.92	0.50	[-1.91, 0.07]	-1.83	0.069
	Combined	Partner Support	-1.08	0.61	[-2.28, 0.12]	-1.77	0.178
		Family Support	-2.18	0.56	[-3.29, -1.08]	-3.89	< 0.001***
		Friend Support	-0.14	0.51	[-1.14, 0.86]	-0.27	0.784

*b* = unstandardised coefficient, SE = standard error, CI = confidence interval. All models included random intercepts for participant ID. Stage group (Trimester 1 - PP Late) was entered as a covariate. Analyses based on aggregated stage-level means from *N* = 331 (EPDS) and *N* = 330 (PASS) observations; not all participants contributed data at all six perinatal stages. Asterisks denote significance: *p* < .05*, *p* < .01**, *p* < .001***

#### Moderation by perinatal stage

**Fig. 1:**
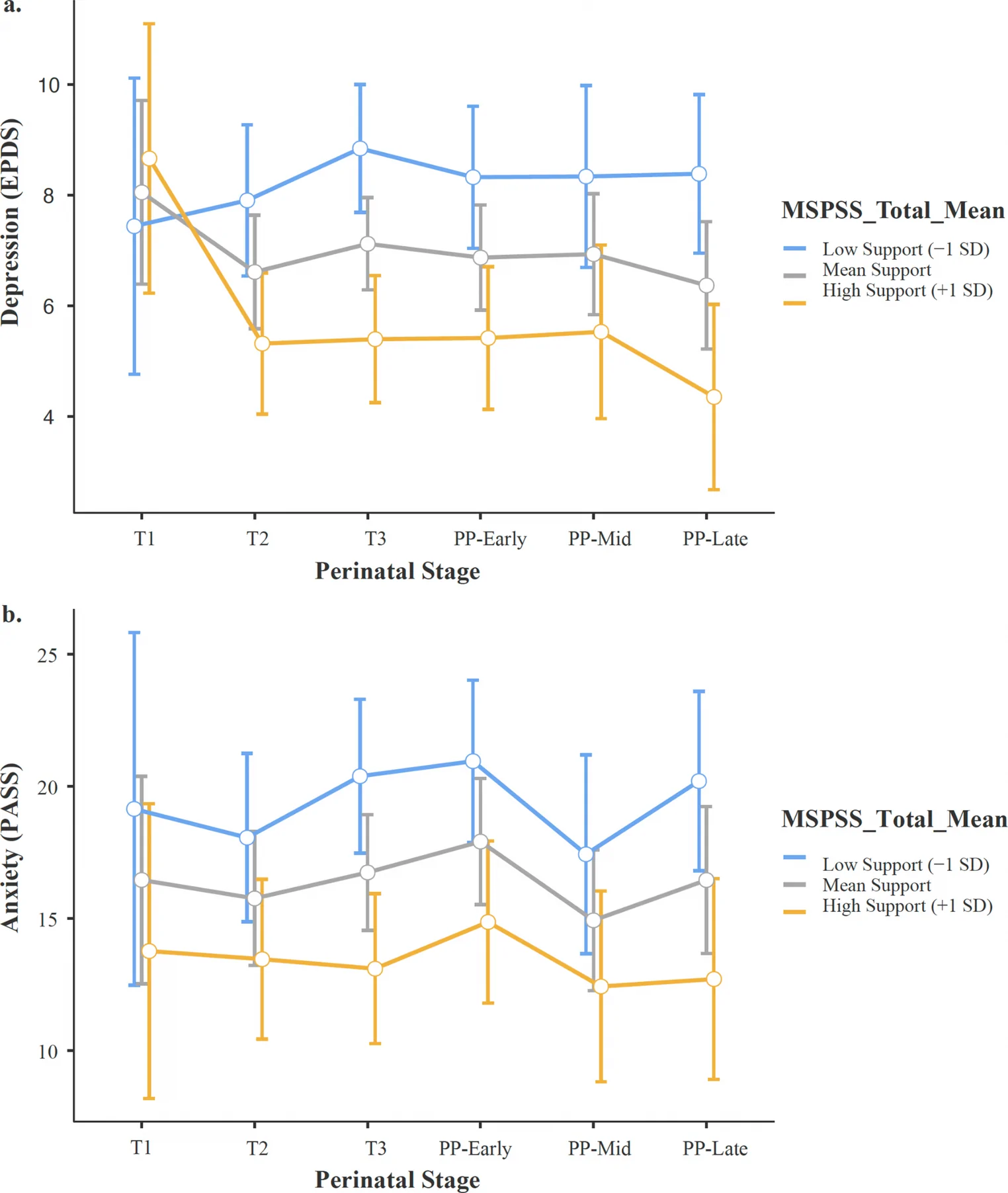
Predicted depression (**a**) and anxiety (**b**) scores across perinatal stages at low (-1 SD), mean, and high (+ 1 SD) levels of total perceived social support Note. Stages are plotted in chronological order. The model used Trimester 3 (T3) as the reference category for fixed effects

Preliminary models including perinatal stage only indicated no overall differences in EPDS or PASS scores across stages. In moderation models including total perceived support and stage, support remained negatively associated with both outcomes, while Stage × Support interactions were not significant (all *p* > .17). Predicted trajectories across stages at low, mean, and high support levels were approximately parallel (Fig. 1). Fixed effects are provided in Table 3.

**Table 3:** Fixed effects from linear mixed models predicting maternal depression and anxiety by perinatal stage and social support

Outcome	Predictor	*b*	SE	95% CI [Lower, Upper]	*t*	*p*
EPDS	Intercept	7.05	0.34	[6.39, 7.72]	20.97	< 0.001***
	Total Support	-1.33	0.34	[-2.01, -0.65]	-3.86	< 0.001***
	Stage	-	-	-	-	ns
	Stage × Support	-	-	-	-	ns
PASS	Intercept	16.38	0.96	[14.50, 18.27]	17.14	< 0.001***
	Total Support	-3.69	0.83	[-5.33, -2.06]	-4.45	< 0.001***
	Stage	-	-	-	-	ns
	Stage × Support	-	-	-	-	ns

*ns =* non-significant omnibus F-tests for stage and interaction terms. All models include random intercepts for participant ID and grand-mean centred predictors. Perinatal stage is coded as six timepoints from Trimester 1 to late postpartum. Analyses based on aggregated stage-level means from *N* = 331 (EPDS) and *N* = 330 (PASS) observations; not all participants contributed data at all six perinatal stages. Asterisks denote significance: *p* < .05*, *p* < .01**, *p* < .001***

#### Moderation by stressors and sleep

**Fig. 2:**
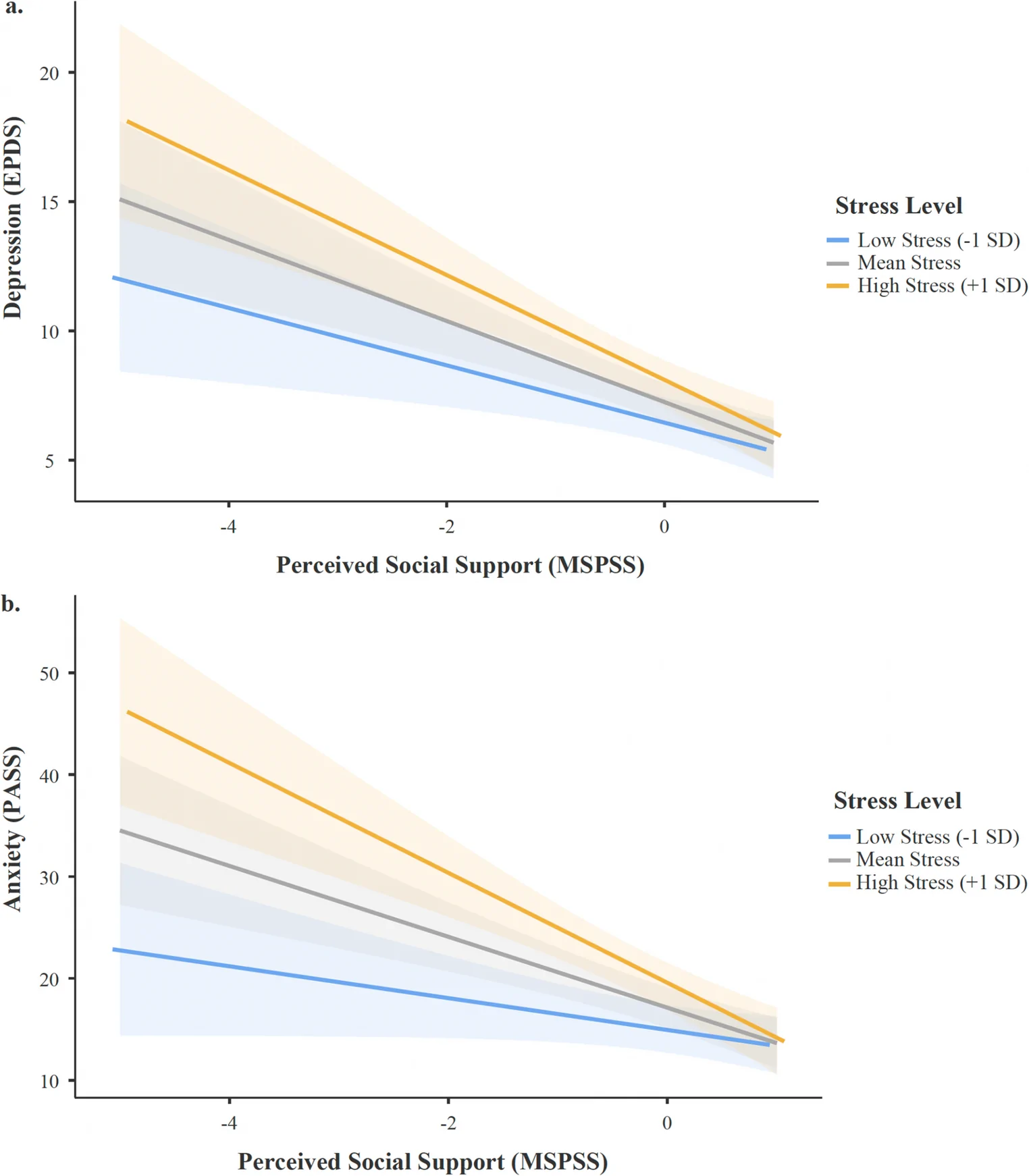
Interaction between perceived social support and stress in predicting depression and anxiety

Cumulative stressors were positively associated with both depression and anxiety scores. The interaction between social support and stressors approached significance for depressive symptoms and was significant for anxiety, indicating that higher perceived social support attenuated the association between cumulative stressors and anxiety symptoms (Fig. 2). Although an omnibus stage effect emerged for anxiety, stage did not interact with social support, and trajectories remained stable across pregnancy and postpartum.

Sleep problems were associated with higher depressive and anxiety symptoms, with no significant Support × Sleep interactions. Full moderation model estimates are provided in Table 4.

**Table 4:** Linear mixed models testing moderation of social support effects on maternal depression and anxiety

Outcome	Moderator	Interaction Term	*b*	SE	95% CI [Lower, Upper]	*t*	*p*
EPDS	Stress		0.56	0.24	[0.09, 1.03]	2.36	0.019*
	Sleep Problems		2.25	0.43	[1.40, 3.10]	5.23	< 0.001***
	Stress	Support × Stress	-0.37	0.20	[-0.76, 0.02]	-1.86	0.064
	Sleep Problems	Support × Sleep	-0.08	0.51	[-1.08, 0.91]	-0.15	0.878
PASS	Stress		1.98	0.54	[0.92, 3.04]	3.67	< 0.001***
	Sleep Problems		3.34	0.96	[1.46, 5.23]	3.49	< 0.001***
	Stress	Support × Stress	-1.58	0.44	[-2.45, -0.71]	-3.59	< 0.001***
	Sleep Problems	Support × Sleep	-0.93	1.14	[-3.18, 1.31]	-0.82	0.414

*b* = unstandardised coefficient, SE = standard error, CI = confidence interval. Reported estimates are from linear mixed models testing stress and sleep problems as moderators of the association between perceived social support (MSPSS total mean) and maternal depression (EPDS) and anxiety (PASS). Models included random intercepts for participant ID and grand-mean centred predictors. Perinatal stage group (Trimester 1 to late postpartum) was included as a covariate. Analyses were based on aggregated stage-level means from *N* = 331 (EPDS) and *N* = 330 (PASS) observations; not all participants contributed data at all stages. Asterisks denote significance: *p* < .05*, *p* < .01**, *p* < .001***

##### Note

Plots show the interaction between total perceived social support (MSPSS) and cumulative stress in predicting (a) depression (EPDS) and (b) anxiety (PASS). MSPSS scores were grand-mean centred, such that negative values reflect lower-than-average perceived social support. Moderation was plotted at low (-1 SD), mean, and high (+ 1 SD) levels of stress. Shaded bands represent 95% confidence intervals.

#### Lagged models

Lagged models examined whether perceived support predicted subsequent distress. Lagged support predicted lower subsequent EPDS scores. In contrast, lagged support did not significantly predict subsequent anxiety. Full lagged estimates are provided in Table S4 ​(Online Resource 1).

## Discussion

This study found that higher perceived social support was associated with lower maternal depression and anxiety across pregnancy and postpartum in Aotearoa New Zealand. Family support emerged as the most consistent protective factor, social support buffered the effects of stress on anxiety, and higher perceived support predicted subsequent reductions in depressive symptoms.

Findings reinforce social support as a robust protective factor across pregnancy and postpartum. While some studies suggest effects are limited to specific periods (Li et al. 2017; Seymour-Smith et al. 2021), others show that antenatal support predicts later outcomes (Bilszta et al. 2008; Milgrom et al. 2008). Evidence for buffering emerged in moderation analyses involving stress, particularly for anxiety, suggesting that anxiety symptoms may be more sensitive to contextual pressures. This highlights the importance of considering anxiety alongside depression in perinatal research and intervention.

A key contribution was the identification of family support as the most stable predictor of reduced maternal distress. When all sources were modelled together, only family support remained significant. This aligns with international evidence highlighting the importance of extended family (Cho et al. 2022; Lee et al. 2023; Reid and Taylor 2015). Family support remained consistently protective despite the emphasis on partner support in Western research (Bilszta et al. 2008; Milgrom et al. 2008). These findings also resonate with Māori frameworks that position wellbeing within collective relational systems, where whānau plays a central role (Durie 1994; Mikahere-Hall 2020; Pitama et al. 2017). Interpretation should remain cautious given the underrepresentation of Māori and Pasifika participants.

However, family support is not universally available. Migrants and women who are separated or estranged from family may be more vulnerable when such support is lacking (Alhasanat-Khalil et al. 2018). Unpartnered women or those in unsupportive relationships may rely more on family or community ties, and poor-quality partner support may be more detrimental than having no partner (Bilszta et al. 2008). For LGBTQIA+ parents, chosen whānau and affirming networks often provide essential protection (Leal et al. 2021), yet are not captured by standardised measures such as the MSPSS.

Stress was a consistent predictor of maternal depression and anxiety, with higher stressor exposure associated with elevated symptoms. Social support moderated these associations differently across outcomes, with a clear buffering effect for anxiety and weaker evidence for depression. This extends previous findings of inconsistent moderation effects (Racine et al. 2019; Reid and Taylor 2015; Yu et al. 2021).

Sleep problems were also associated with elevated distress, consistent with existing literature (Okun et al. 2018; Signal et al. 2022). However, sleep did not moderate the effects of perceived social support. Sleep may contribute directly to maternal distress rather than modifying the protective effects of support. Recent research suggests these relationships are complex and may operate through multiple pathways (Wang et al. 2024; Dai et al. 2026). This finding may also reflect the type of support assessed. The MSPSS primarily measures perceived emotional support, whereas perinatal sleep may be more closely related to instrumental or practical support, such as assistance with infant care or opportunities for uninterrupted rest. This interpretation is consistent with qualitative research highlighting the importance of instrumental support for postpartum recovery (Negron et al. 2013).

Lagged analyses indicated that higher perceived support predicted lower subsequent depressive symptoms but not subsequent anxiety, suggesting that depression may reflect longer-term relational patterns, whereas anxiety appears more reactive to immediate stressors. These findings support strengthening social support early in the perinatal period.

Several limitations should be considered. The sample was relatively homogeneous, comprising predominantly highly educated, partnered women, with underrepresentation of Māori and Pasifika mothers, limiting generalisability. Although attrition was non-random, with higher baseline distress predicting dropout, linear mixed models appropriately accommodate incomplete repeated-measures data under the MAR assumption; however, bias cannot be excluded if attrition was also influenced by unmeasured factors. Moderation findings primarily reflect the main sample because stressors were not measured in the pilot cohort.

Ceiling effects limited the explanatory power of partner support, anxiety was measured less frequently than depression, and the MSPSS does not capture professional or community-based support or relationship quality.

Findings reinforce the importance of social support, particularly family support, as a key protective factor for maternal mental health. Clinical practice should routinely assess the availability and quality of family support while recognising alternative sources of support where needed. Screening for stressors and sleep difficulties may identify women requiring additional support, while sleep difficulties should be addressed alongside efforts to strengthen social support.

Family-inclusive approaches across antenatal and postpartum care should remain responsive to diverse family structures, including single mothers, migrants, and LGBTQIA+ parents. Although the sample was predominantly Pākehā, the prominence of family support aligns with Māori frameworks that position whānau as central to wellbeing, suggesting broader relevance of relational models.

Overall, this study provides longitudinal evidence that perceived social support, particularly family support, protects maternal mental health across pregnancy and postpartum in Aotearoa New Zealand, supporting family-centred, contextually responsive approaches to perinatal care.

## Supplementary information

Below is the link to the electronic supplementary material.

Supplementary Material 1

## Data Availability

The data that support the findings of this study are not publicly available due to ethical and privacy restrictions but may be available from the corresponding author on reasonable request, subject to approval by the original study investigators and relevant ethics committees.
