# Supplementary material for: Longitudinal associations between perceived social support and maternal depression and anxiety across the perinatal period
Source: Arch Womens Ment Health. 2026 Nov 14;29(6):150. doi: 10.1007/s00737-026-01785-0 (PMC13769739; doi:10.1007/s00737-026-01785-0)
Supplement: Supplementary file 1 [file 737_2026_1785_MOESM1_ESM.docx]

## **Online Resource 1**

**Table S1**
*Mean scores (SD) for depression, anxiety, perceived social support, sleep problems, and stressors by perinatal stage*

| **Stage** | **EPDS *M* (*SD*)** | **PASS *M* (*SD*)** | **MSPSS Total *M* (*SD*)** | **MSPSS Partner *M* (*SD*)** | **MSPSS Family *M* (*SD*)** | **MSPSS Friends *M* (*SD*)** | **Sleep problems *M* (*SD*)** | **Stressor count *M* (*SD*)** |
| --- | --- | --- | --- | --- | --- | --- | --- | --- |
| Trimester 1 | 7.60 (4.51) | 14.8 (10.0) | 6.05 (0.76) | 6.59 (0.75) | 5.92 (1.10) | 5.65 (1.36) | 0.45 (0.51) | 0.75 (1.22) |
| Trimester 2 | 6.64 (4.20) | 15.4 (11.0) | 6.11 (0.97) | 6.50 (1.03) | 5.93 (1.36) | 5.90 (1.08) | 0.27 (0.45) | 0.95 (1.06) |
| Trimester 3 | 7.18 (4.17) | 16.4 (10.9) | 6.04 (0.83) | 6.47 (0.80) | 5.92 (1.07) | 5.74 (1.15) | 0.47 (0.50) | 1.04 (1.22) |
| Early postpartum | 6.67 (4.71) | 16.9 (14.7) | 6.03 (0.89) | 6.44 (0.79) | 5.87 (1.33) | 5.77 (1.14) | 0.35 (0.48) | 0.91 (1.09) |
| Mid postpartum | 7.12 (4.36) | 14.7 (10.4) | 6.07 (0.73) | 6.58 (0.68) | 6.00 (1.05) | 5.63 (1.21) | 0.31 (0.46) | 1.05 (1.21) |
| Late postpartum | 5.78 (4.69) | 13.4 (9.35) | 5.83 (0.83) | 6.30 (0.82) | 5.98 (0.94) | 5.21 (1.36) | 0.31 (0.41) | 0.98 (1.11) |

*Note.* Means and standard deviations are based on aggregated stage-level means from *N* = 331 (EPDS) and *N* = 330 (PASS) observations; not all participants contributed data at all six perinatal stages. Sleep problems were coded 0 = no problem, 1 = problem. Higher EPDS and PASS scores indicate greater distress. MSPSS scores range from 1–7, with higher scores indicating stronger perceived support. Stress count represents the number of stressful life events reported at each stage.

**Table S2a**

*Pearson correlations between social support and depression (EPDS) by perinatal stage*

| **Stage** | **Partner** | **Family** | **Friends** | **Total** |
| --- | --- | --- | --- | --- |
| Trimester 1 | -0.201 | 0.094 | -0.082 | -0.074 |
| Trimester 2 | -0.275* | -0.373* | -0.297* | -0.382** |
| Trimester 3 | -0.317*** | -0.348*** | -0.361*** | -0.420*** |
| Early Postpartum | -0.309* | -0.457*** | -0.199 | -0.404*** |
| Mid Postpartum | -0.103 | -0.450** | -0.226 | -0.373** |
| Late Postpartum | -0.378** | -0.442** | -0.209 | -0.408** |

*Note.* All coefficients are Pearson’s *r*. Asterisks denote significance: *p* < .05*, *p* < .01**, *p* < .001***. ‘Partner’ refers to MSPSS Significant Other subscale. Correlations are based on aggregated stage-level means from *N* = 331 (EPDS) and *N* = 330 (PASS) observations; not all participants contributed data at all six perinatal stages.

**Table S2b**

*Pearson correlations between social support and anxiety (PASS) by perinatal stage*

| **Stage** | **Partner** | **Family** | **Friends** | **Total** |
| --- | --- | --- | --- | --- |
| Trimester 1 | -0.557* | -0.350 | -0.309 | -0.540* |
| Trimester 2 | -0.260 | -0.469*** | -0.162 | -0.370* |
| Trimester 3 | -0.211* | -0.353*** | -0.440*** | -0.426*** |
| Early Postpartum | -0.296* | -0.473*** | -0.141 | -0.383** |
| Mid Postpartum | -0.091 | -0.442** | -0.242 | -0.373** |
| Late Postpartum | -0.285* | -0.473*** | -0.006 | -0.275 |

*Note.* All coefficients are Pearson’s *r*. Asterisks denote significance: *p* < .05*, *p* < .01**, *p* < .001***. ‘Partner’ refers to MSPSS Significant Other subscale. Correlations are based on aggregated stage-level means from *N* = 331 (EPDS) and *N* = 330 (PASS) observations; not all participants contributed data at all six perinatal stages.

**Table S3a**

*Bivariate Pearson correlations between psychological distress and demographic and psychosocial covariates at each perinatal stage group*
*Correlation matrix - trimester 1*

| **Variable** | **EPDS** | **PASS** | **Stress Count** | **Sleep Problems** | **Education** | **Age** |
| --- | --- | --- | --- | --- | --- | --- |
| Depression (EPDS) | - |  |  |  |  |  |
| Anxiety (PASS) | .455* | - |  |  |  |  |
| Stress count | .375 | .799** | - |  |  |  |
| Sleep problems | -.192 | -.069 | .000 | - |  |  |
| Education | -.464 | -.720* | -.615* | -.239 | - |  |
| Age | -.129 | -.582** | -.598* | .283 | .577 | - |

*Note.* Values are Pearson’s *r* (two-tailed). Asterisks denote significance: *p* < .05*, *p* < .01**, *p* < .001***. Correlations are based on aggregated stage-level means from *N* = 331 (EPDS) and *N* = 330 (PASS) observations; not all participants contributed data at all six perinatal stages.

**Table S3b**
*Correlation matrix - trimester 2*

| **Variable** | **EPDS** | **PASS** | **Stress Count** | **Sleep Problems** | **Education** | **Age** |
| --- | --- | --- | --- | --- | --- | --- |
| Depression (EPDS) | - |  |  |  |  |  |
| PASS (Anxiety) | .692*** | - |  |  |  |  |
| Stress count | .275 | .364* | - |  |  |  |
| Sleep problems | .367** | .230 | .203 | - |  |  |
| Education | -.205 | -.320* | -.045 | -.040 | - |  |
| Age | -.102 | -.295* | .148 | .197 | .511** | - |

*Note.* Values are Pearson’s *r* (two-tailed). Asterisks denote significance: *p* < .05*, *p* < .01**, *p* < .001***. Correlations are based on aggregated stage-level means from *N* = 331 (EPDS) and *N* = 330 (PASS) observations; not all participants contributed data at all six perinatal stages.

**Table S3c**
*Correlation matrix - trimester 3*

| **Variable** | **EPDS** | **PASS** | **Stress Count** | **Sleep Problems** | **Education** | **Age** |
| --- | --- | --- | --- | --- | --- | --- |
| Depression (EPDS) | - |  |  |  |  |  |
| PASS (Anxiety) | .637*** | - |  |  |  |  |
| Stress count | .208 | .514*** | - |  |  |  |
| Sleep problems | .126 | .116 | .238* | - |  |  |
| Education | -.196 | -0.337** | -.242* | -.063 | - |  |
| Age | -.167 | -.275* | -.249* | .041 | .509*** | - |

*Note.* Values are Pearson’s *r* (two-tailed). Asterisks denote significance: *p* < .05*, *p* < .01**, *p* < .001***. Correlations are based on aggregated stage-level means from *N* = 331 (EPDS) and *N* = 330 (PASS) observations; not all participants contributed data at all six perinatal stages.

**Table S3d**
*Correlation matrix - early postpartum*

| **Variable** | **EPDS** | **PASS** | **Stress Count** | **Sleep Problems** | **Education** | **Age** |
| --- | --- | --- | --- | --- | --- | --- |
| Depression (EPDS) | - |  |  |  |  |  |
| PASS (Anxiety) | .822*** | - |  |  |  |  |
| Stress count | .427** | .549*** | - |  |  |  |
| Sleep problems | .459*** | .337** | .173 | - |  |  |
| Education | -.233 | -.245 | -.262 | -.108 | - |  |
| Age | -.397** | -.485*** | -.343* | -.043 | 0.379** | - |

*Note.* Values are Pearson’s *r* (two-tailed). Asterisks denote significance: *p* < .05*, *p* < .01**, *p* < .001***. Correlations are based on aggregated stage-level means from *N* = 331 (EPDS) and *N* = 330 (PASS) observations; not all participants contributed data at all six perinatal stages.

**Table S3e**
*Correlation matrix - mid postpartum*

| **Variable** | **EPDS** | **PASS** | **Stress Count** | **Sleep Problems** | **Education** | **Age** |
| --- | --- | --- | --- | --- | --- | --- |
| Depression (EPDS) | - |  |  |  |  |  |
| PASS (Anxiety) | .65*** | - |  |  |  |  |
| Stress count | .417** | .466** | - |  |  |  |
| Sleep problems | .340* | .019 | -.084 | - |  |  |
| Education | -.193 | -.153 | -.108 | .162 | - |  |
| Age | -.149 | -.388** | -.308 | .100 | .332* | - |

*Note.* Values are Pearson’s *r* (two-tailed). Asterisks denote significance: *p* < .05*, *p* < .01**, *p* < .001***. Correlations are based on aggregated stage-level means from *N* = 331 (EPDS) and *N* = 330 (PASS) observations; not all participants contributed data at all six perinatal stages.

**Table S3f**
*Correlation matrix - late postpartum*

| **Variable** | **EPDS** | **PASS** | **Stress Count** | **Sleep Problems** | **Education** | **Age** |
| --- | --- | --- | --- | --- | --- | --- |
| Depression (EPDS) | - |  |  |  |  |  |
| PASS (Anxiety) | .777*** | - |  |  |  |  |
| Stress count | .495*** | .556*** | - |  |  |  |
| Sleep problems | .445** | .246 | .342* | - |  |  |
| Education | -.173 | -.219 | -.368* | .186 | - |  |
| Age | -.136 | -.330* | -.031 | -.147 | .192 | - |

*Note.* Values are Pearson’s *r* (two-tailed). Asterisks denote significance: *p* < .05*, *p* < .01**, *p* < .001***. Correlations are based on aggregated stage-level means from *N* = 331 (EPDS) and *N* = 330 (PASS) observations; not all participants contributed data at all six perinatal stages.

**Table S4**

*Significant fixed effects from lagged linear mixed models predicting depression (EPDS) and anxiety (PASS)*

| **Predictor** | ***b* (EPDS)** | **SE (EPDS)** | ***p* (EPDS)** | ***b* (PASS)** | **SE (PASS)** | ***p* (PASS)** |
| --- | --- | --- | --- | --- | --- | --- |
| Intercept | 6.14 | 0.41 | <.001*** | 15.19 | 1.22 | <.001*** |
| Survey Month 3 | -1.09 | 0.53 | .041* | - | - | - |
| Survey Month 12 | -2.41 | 1.01 | .018* | - | - | - |
| Lagged MSPSS | -1.47 | 0.37 | <.001*** | -1.08 | 0.88 | .220 |

*Note.* *b* = unstandardised coefficient; SE = standard error. Survey month contrasts represent estimated differences from baseline (Month 0). Lagged predictors tested whether perceived social support (MSPSS total mean at survey t) predicted distress (EPDS, PASS) at the subsequent survey wave (t+1). Only statistically significant fixed effects (*p* < .05) are presented. Models included random intercepts for participant ID; predictors were grand-mean centred. Asterisks denote significance: *p* < .05*, *p* < .01**, *p* < .001***
